# Supplementary material for: Remote-controlled mechanical and directional motions of photoswitchable DNA condensates
Source: Nat Commun. 2025 May 14;16:4479. doi: 10.1038/s41467-025-59100-x (PMC12078559; doi:10.1038/s41467-025-59100-x)
Supplement: Supplementary file 1 — Supplementary Information [file 41467_2025_59100_MOESM1_ESM.pdf]

Remote-controlled mechanical and directional motions of photoswitchable DNA condensates

Hirotake Udono[1], Shin-ichiro M. Nomura[2], Masahiro Takinoue[1,3*]

1 Department of Computer Science, School of Computing, Institute of Science Tokyo, Yokohama, Kanagawa, 226-8501, Japan.

2 Department of Robotics, Graduate School of Engineering, Tohoku University, Sendai, Miyagi, 980-8579, Japan.

3 Research Center for Autonomous Systems Materialogy (ASMat), Institute of Innovative Research (IIR), Institute of Science Tokyo, Yokohama, Kanagawa, 226-8501, Japan.

* E-mail: takinoue@comp.isct.ac.jp

Table of Contents

• Supplementary Texts, Tables, and Figures

1. Sequence design
2. Light irradiation for native PAGE
3. Reconstruction of DNA condensates with UV-to-Vis switching
4. FRAP experiments for photoresponsive DNA
5. Native PAGE
6. Initial configurations in cross-linked motif condensates
7. Microflow reversibility in photoswitching
8. Surface stress effects in UV-induced phase-state changes
9. Measurement of MSD
10. Evaluation of flow mobility from MSD
11. Enthalpy estimation of Azo-modified SEs
12. Directional motion of DNA liquid-state condensates subjected to localized photoswitching
13. Numerical experiments of the directional motion in localized photoswitching

• Supplementary References

Supplementary Text, Tables, and Figures

1. Sequence design

- Y-shaped DNA nanostructures (Y-motifs)

In the following tables, sticky ends (SEs) are marked by bold fonts. Sequences marked in the same color form a double-stranded stem.

Supplementary Table 1 Y-motif without azobenzene (Y)

Name	Sequence (5'–3')
Y-1	**GCTCGAGC**CAGTGAGGACGGAAGTTTGTCGTAGCATCGCACC
Y-2	**GCTCGAGC**CAACCACGCCTGTCCATTACTTCCGTCCTCACTG
Y-3	**GCTCGAGC**GGTGCGATGCTACGACTTTGGACAGGCGTGGTTG

Sequences were referenced from Y. Sato *et al.* (2020).

Supplementary Table 2 Y-motif with azobenzene (Y_{1x7})

Name	Sequence (5'–3')
Y_{1x7}-1	**GxCTCGAGC**...
Y_{1x7}-2	**GxCTCGAGC**...
Y_{1x7}-3	**GxCTCGAGC**...

Azobenzene ('x') was inserted in the SEs of Y. Stem sequences (...) were the same as the counterparts of Y.

Supplementary Table 3 Y-motif with azobenzene (Y_{3x5})

Name	Sequence (5'–3')
Y_{3x5}-1	**GCTxCGAGC**...
Y_{3x5}-2	**GCTxCGAGC**...
Y_{3x5}-3	**GCTxCGAGC**...

Supplementary Table 4 Y-motif with azobenzene (Y_{2x1x5})

Name	Sequence (5'–3')
Y_{2x1x5}-1	**GCxTxCGAGC**...
Y_{2x1x5}-2	**GCxTxCGAGC**...
Y_{2x1x5}-3	**GCxTxCGAGC**...

Supplementary Table 5 Y-motif with azobenzene (Y_{2x4})

Name	Sequence (5'–3')
Y_{2x4}-1	GCxTAGC...
Y_{2x4}-2	GCxTAGC...
Y_{2x4}-3	GCxTAGC...

Supplementary Table 6 Y-motif with a single SE (Control)

Name	Sequence (5'–3')
Y'-1	(The same as Y-1)
Y'-2	CAACCACGCCTGTCCATTACTTCCGTCCTCACTG
Y'-3	GGTGCGATGCTACGACTTTGGACAGGCGTGGTTG

Supplementary Table 7 Azobenzene-tethered Y-motif with a single SE (SE_{1x7})

Name	Sequence (5'–3')
Y'_{1x7}-1	(The same as Y_{1x7}-1)
Y'_{1x7}-2	(The same as Y'-2)
Y'_{1x7}-3	(The same as Y'-3)

Supplementary Table 8 Azobenzene-tethered Y-motif with a single SE (SE_{3x5})

Name	Sequence (5'–3')
Y'_{3x5}-1	(The same as Y_{3x5}-1)
Y'_{3x5}-2	(The same as Y'-2)
Y'_{3x5}-3	(The same as Y'-3)

Supplementary Table 9 Azobenzene-tethered Y-motif with a single SE (SE_{2x1x5})

Name	Sequence (5'–3')
Y'_{2x1x5}-1	(The same as Y_{2x1x5}-1)
Y'_{2x1x5}-2	(The same as Y'-2)
Y'_{2x1x5}-3	(The same as Y'-3)

- Cross-linked DNA motifs for sequence-specific photo-responsiveness

Supplementary Table 10 Cross-linked DNA systems ($Y_i/L_0/Y_0$, i = 1x7, 3x5, 2x1x5)

Name	Sequence (5'–3')
Y_i-1	(The same as Y_i-1, i = 1x7, 3x5, 2x1x5)
Y_i-2	(The same as Y_i-2, i = 1x7, 3x5, 2x1x5)
Y_i-2_FAM	[FAM]-CAACCACGCCTGTCCATTACTTCCGTCCTCACTG
Y_i-3	(The same as Y_i-3, i = 1x7, 3x5, 2x1x5)
Y_0-1	**CTCGCGAG**AAAGGAACTCTCCGCGTTGACAAAGCCGACACGT
Y_0-2	**CTCGCGAG**GCCTCTGTGTCGCATCTTCGCGGAGAGTTCCTTT
Y_0-2_Alexa405	[Alexa405]-GCCTCTGTGTCGCATCTTCGCGGAGAGTTCCTTT
Y_0-2_Cy3	[Cy3]-GCCTCTGTGTCGCATCTTCGCGGAGAGTTCCTTT
Y_0-3	**CTCGCGAG**ACGTGTCGGCTTTGTCTTGATGCGACACAGAGGC
L_0-1	**CTCGCGAG**GCTGGACTAACGGAACGGTTAGTCAGGTATGCCAGCAC
L_0-2	**CTCGCGAG**CTCAGAGAGGTGACAGCATTCCGTTCCGTTAGTCCAGC
L_0-3	**CTCGCGAG**CCATGGTCCCAAGTGATGTTTGCTGTCACCTCTCTGAG
L_0-4	**GCTCGAGC**CGGCGCTGTAAATTTGCGTTCATCACTTGGGACCATGG
L_0-5	**GCTCGAGC**CAGACGTCACTCTCCAACTTCGCAAATTTACAGCGCCG
L_0-6	**GCTCGAGC**GTGCTGGCATACCTGACTTTGTTGGAGAGTGACGTCTG

- Plain Y motifs with redesigned SEs

Supplementary Table 11 rY_A

Name	Sequence (5'–3')
rY_A-1	**ATTATAAT**...
rY_A-2	**ATTATAAT**...
rY_A-3	**ATTATAAT**...

The stem sequences (...) were the same as the counterparts of Y.

Supplementary Table 12 rY_B

Name	Sequence (5'–3')
rY_B-1	**TTCGAA**...
rY_B-2	**TTCGAA**...
rY_B-3	**TTCGAA**...

The stem sequences (...) were the same as the counterparts of Y.

Supplementary Table 13 rY_C

Name	Sequence (5′–3′)
rY_C-1	GATATATC...
rY_C-2	GATATATC...
rY_C-3	GATATATC...

Supplementary Table 14 rY_D

Name	Sequence (5′–3′)
rY_D-1	ACTTAAGT...
rY_D-2	ACTTAAGT...
rY_D-3	ACTTAAGT...

Supplementary Table 15 rY_E

Name	Sequence (5′–3′)
rY_E-1	GATCGATC...
rY_E-2	GATCGATC...
rY_E-3	GATCGATC...

Supplementary Table 16 rY_F

Name	Sequence (5′–3′)
rY_F-1	GACTCGAGTC...
rY_F-2	GACTCGAGTC...
rY_F-3	GACTCGAGTC...

Sequences were referenced from Y. Sato *et al.* (2020).

Supplementary Table 17 rY_G

Name	Sequence (5′–3′)
rY_G-1	GCTAGCGCTAGC...
rY_G-2	GCTAGCGCTAGC...
rY_G-3	GCTAGCGCTAGC...

Sequences were referenced from Y. Sato *et al.* (2020).

- Cross-linked DNA motifs for cargo transport with directional motion

Supplementary Table 18 Cross-linked DNA system (Y_{2x4}/L'_0/Y_0)

Name	Sequence (5'–3')
Y_{2x4}-1,2,3	(See above)
Y_{2x4}-2_FAM	(The same as Y_i-2_FAM, i = 1x7, 3x5, 2x1x5)
Y_0-1,2,3; Y_0-2_Cy3	(See above)
L'_0-1,2,3	(The same as L_0-1,-2,-3, respectively)
L'_0-4	**GCTAGC**...
L'_0-5	**GCTAGC**...
L'_0-6	**GCTAGC**...

The stem sequences (...) were the same as the counterparts of L_0.

2. Light irradiation for native PAGE

Before irradiation in PAGE experiments, solutions of single-SE Y-motif were sucked by UV-transmissive glass capillaries, a fragment snapped off from a glass tubing (World Precision Instrument) with a proper length. The capillaries were then placed on a black metal plate during photo-irradiation to prevent visible light (Vis) reflection of UV light and thermal radiation.

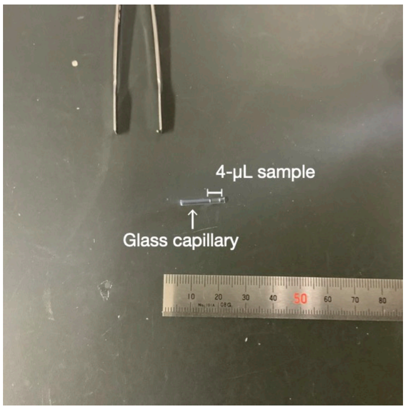

Supplementary Fig. 1 Image of irradiation materials for PAGE experiments. Before photo-irradiation, a sample of single-SE Y-motif (Supplementary Table 8) was sucked by a glass capillary.

3. Reconstruction of DNA condensates with UV-to-Vis switching

We have shown the photoreversible phase-state changes of DNA condensates in Figs. 2a, b. To examine whether the Vis-reconstructed DNA condensates could recover to the original sizes, we performed particle size analysis at varied Vis irradiation times (Supplementary Fig. 2). Initially, DNA condensates were dissociated by UV irradiation before switching to Vis. We observed a distinct growth of the recondensed DNA in the size within the initial 20 min, whereas no further significant growth was observed beyond ~1 h. As previously explored, the growth of phase-separated DNA liquid condensates starts with fast nucleation, followed by countless coalescence events, rather than Ostwald ripening[1]. Photoswitched DNA motifs under isothermal conditions undergo less frequent encounters with each other than under thermal annealing, which entails incessant thermal fluctuations at higher temperature ranges. The isothermal conditions may have led to the inefficient recondensation in UV-to-Vis switching. In addition, at the applied temperature of 53°C, thermal dissociation might also have been non-negligible and counteracted the condensate growth in the recondensation process.

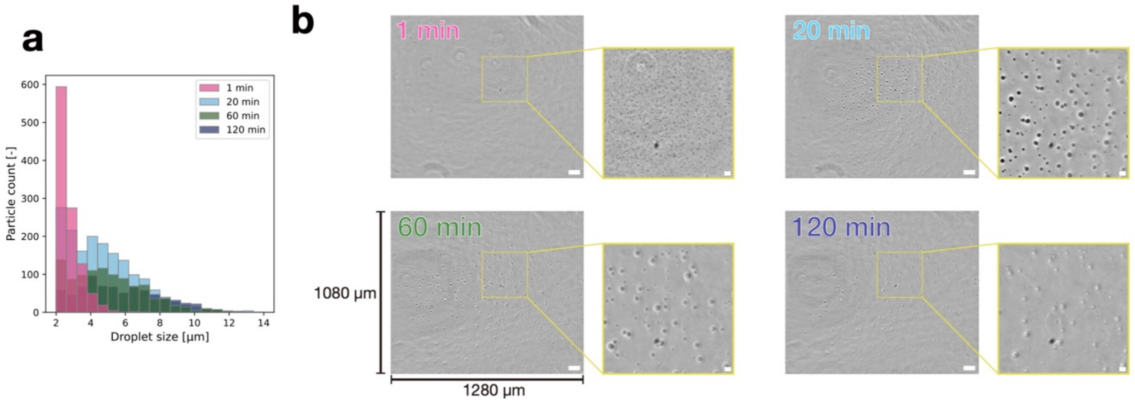

Supplementary Fig. 2 Particle size analysis of DNA condensates undergoing a dissociated-to-liquid state change by Vis irradiation. a, Size distributions as a function of Vis irradiation time. **b**, Representative images using phase-contrast (PC) microscopy of Vis-reconstructed DNA condensates at various elapsed times. Scale bars: 50 μm, (inset) 10 μm.

4. FRAP experiments for photoresponsive DNA

Our confocal microscopy that uses the scanning laser in the visible range (405 nm for photobleaching, 473 nm for fluorophore excitation) affects the phase state of photoresponsive DNA condensates. As in Supplementary Fig. 3a, we conducted photobleaching (30 s) before UV/Vis irradiation (30 s) upon the samples, and ended each experiment when the sample was scanned with the excitation laser. While common FRAP bleaching must be preceded by reactions of interest, we inverted this order in the current FRAP procedures to prevent the bleaching from affecting the SE binding affinity (Fig. 2d). Thus, it is reminded that the fluorescence recovery and photoinduced state change proceeded simultaneously following the photobleaching. For similar reasons, each sample was replaced with a new one after imaging the fluorescence recovery at a specific elapsed time, rather than capturing time-series data, *i.e.*, each data point corresponds to a single sample imaged.

As a result of the post-bleaching irradiation, we noticed that UV irradiation could cause fluorescence degradation in the overall DNA hydrogel particles (see 'UV→Dark' of Fig. 2e). The darkened images might result in an artifact of false decrease in the fluorescence recovery. Thus, besides the bleached region in a gel particle of interest ("LBR"), we arbitrarily selected a non-bleached region ("NBR") as the reference to cancel out the degradation-associated signal decrease (Supplementary Fig. 3b).

Fluorescence recoveries in the captured confocal microscopy images were quantified as follows (Supplementary Fig. 3b, c). First, pixel intensities of LBR (I_{LBR}) and NBR (I_{NBR}) were extracted from three consecutive images in a single FRAP experiment: 'initial' image captured the initial state of condensates before photobleaching; '$t = 0$' was acquired immediately after photobleaching; '$t = x$' was obtained when scanning the sample at a specific time of x [s] after the photobleaching. Second, the ratio of I_{LBR}/I_{NBR} was yielded for each sequence. Finally, the ratio was normalized with the initial I_{LBR}/I_{NBR} value. The normalized I_{LBR}/I_{NBR} values were plotted as a function of elapsed time (Supplementary Fig. 3d).

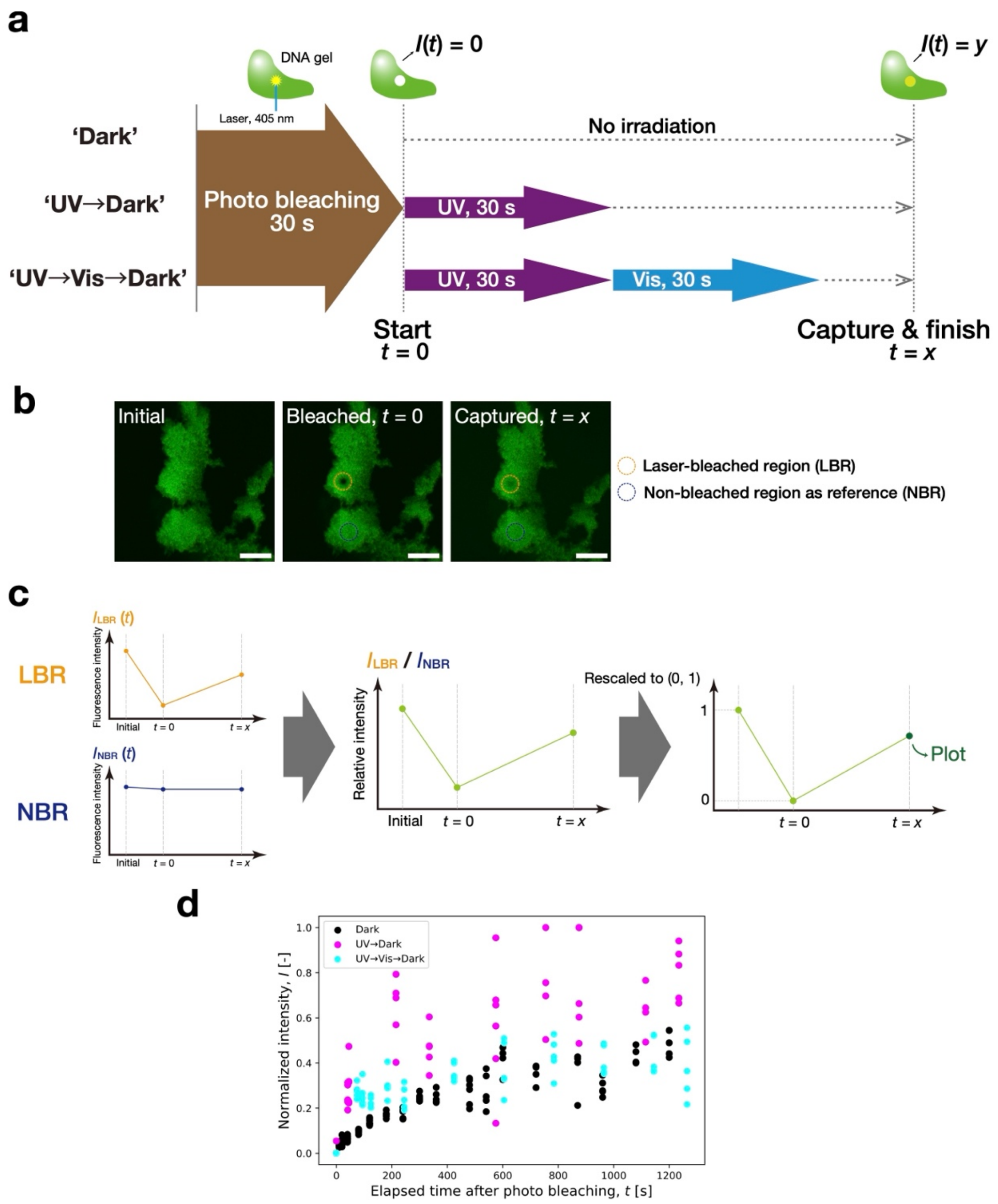

Supplementary Fig. 3 FRAP experiments. **a**, Illustration of the protocol. **b**, Representative sequential images of a FRAP experiment in 'UV' condition. Two dashed-line circles mark a laser-bleached region (orange, "LBR") and a non-bleached region as the reference (dark blue, "NBR"), respectively. Scale bars: 50 µm. **c**, Illustration of the quantification steps in a FRAP experiment. **d**, Scatter plot of the normalized I_{LBR}/I_{NBR} values ($n \geq 4$ at each elapsed time), presenting the individual data points of Fig.2e.

5. Native PAGE

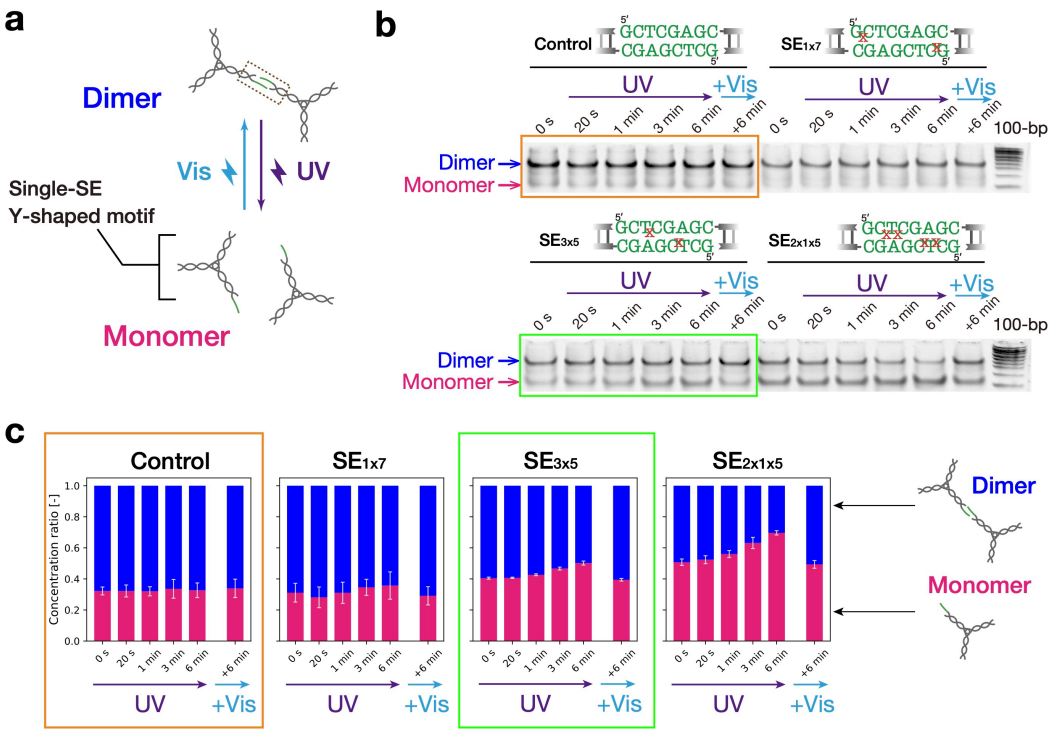

Supplementary Fig. 4 Molecular-level basis for photoswitchable state transition. a, Y-motifs with a single SE were used to generate binary bands in native polyacrylamide gel electrophoresis (PAGE). The upper and lower bands correspond to the dimeric and monomeric states, respectively. **b**, PAGE results at 4°C. Lane labels refer to the durations of UV (0–6 min) and additional Vis (+6 min) irradiation. "100-bp" means 100-bp (base pair) DNA ladder. (Top) Illustration of 8-bp hybridized SEs marked within the dashed-line box in **a**. "x" denotes an inserted Azo. **c**, Bar plots for the concentrations of dimers and monomers measured from the band intensities in **b**. The intensity values in each lane are normalized by the total integrated intensity. Error bars: S.D. ($n = 3$). **b**, **c**, Compare the data enclosed in orange and green boxes as experimental support for the molecular-level photoreversibility as discussed in the main manuscript. Irradiation intensities: 2.0 mW cm^{-2} for UV and 3.9 mW cm^{-2} for Vis. Source data are provided as Source Data files.

6. Initial configurations in cross-linked motif condensates

Below, we present representative snapshots of the initial states of cross-linked DNA condensates at low and high temperatures (Supplementary Fig. 5), in addition to those displayed in Figs. 4, 5. Photoresponsive DNA gel-state condensates (FAM, green) and non-photoresponsive DNA gel-state condensates (Cy3, blue) were adhered well due to the cross-linker DNA, as shown in Fig. 4.

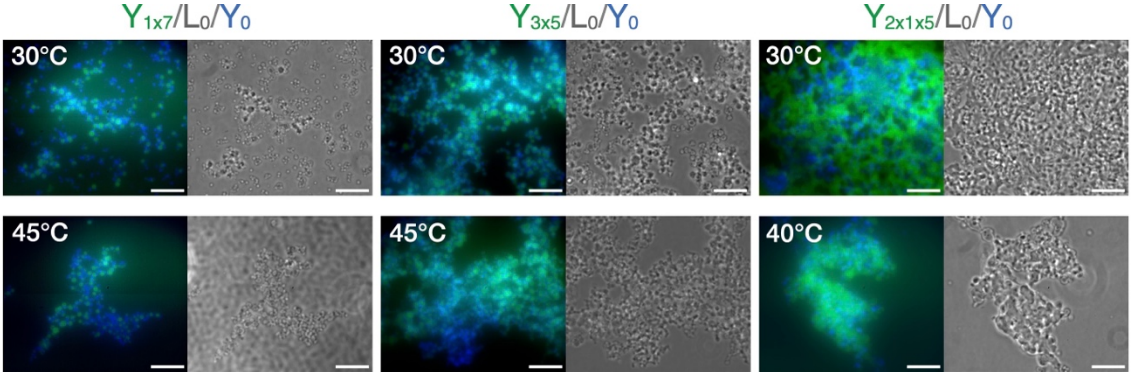

Supplementary Fig. 5 Representative snapshots of the initial configurations of cross-linked DNA condensates. In each cross-linked motif design (row), (left) merged fluorescence images (FAM: green, Cy3: blue) and (right) PC images are displayed for (top) lower and (bottom) higher temperatures. Scale bars: 50 µm.

7. Microflow reversibility in photoswitching

The limitation of the "spread and collect" cycle (Supplementary Fig. 6) can be explained as follows. One undoubtful reason was the unconfined geometry of the observation chamber: a finite fraction of the *cis*-to-*trans* photoswitched Y_{3x5} motifs should have failed to join the reversing flow because of significantly diffusing out into the bulk field. Hence, the use of a more tightly confined environment would improve the collection efficiency. Another mechanism involved was inherent incompleteness in the photoswitchable isomerization of azobenzene residues. Photo-irradiation does not produce 100% efficient *cis–trans* switching due to an overlap between their absorption wavelength spectra[2–4], in contrast to slow but 100% efficient thermal *cis–trans* relaxation in the dark adapation[4,5]. This inherent photo-switching inefficiency inhibits photo-switching cycles.

The asymmetric response speed in the photoswitching cycle reflects the thermodynamic aspect of the flow-generating mechanism. In the UV irradiation, the resulting concentration gradient of dissolved DNA motifs, which drives fast diffusive migration, arises from the deterministic dissociation between SEs. In contrast, in the Vis irradiation, the reversing migration requires the reassociation of SEs, with the binding rate that is largely dependent on frequent random encounters as discussed for the recondensation demonstrated in Fig.2b. Hence, the translation of the SEs' reassociation to the macroscopic reversing flow would be a stochastic process. This thermodynamic difference in the photoswitching cycles must have led to the asymmetric speeds in the expansion/convergence-cycles.

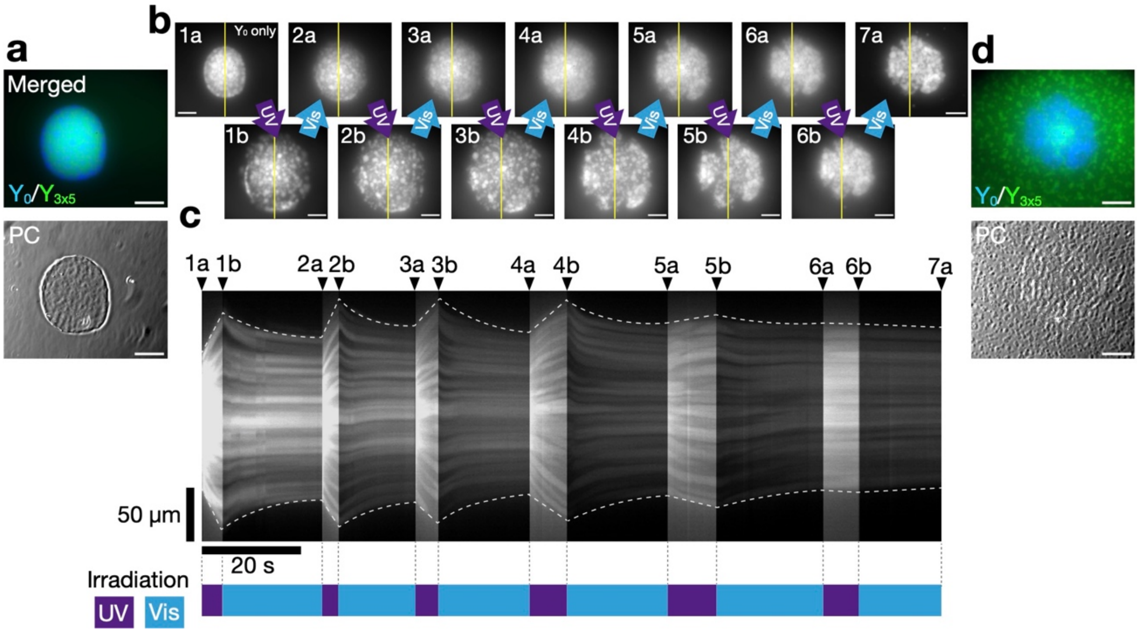

Supplementary Fig. 6 Cyclic microflow of $Y_{3x5}/L_0/Y_0$ liquid condensate under alternating UV/Vis irradiation. a, Florescence (merged) and PC images of a $Y_{3x5}/L_0/Y_0$ liquid condensate at the initial state before the irradiation at 55°C. **b,** Time-sequential snapshots of non-photoresponsive Y_0 phase (labeled with Alexa405/Cy3) in UV–Vis switching. Throughout this photoswitching, **c** a kymograph is acquired along the yellow lines, indicating the alternate dilation (UV) and convergence (Vis) of the Y_0 phase. Photoswitching moments are related to the corresponding snapshots in **b**. Curved dashed lines indicate the outermost interface of the flowing condensate to guide the eye. **d,** Fluorescence (merged) and PC images in the final state after the irradiation. Scale bars: 50 µm.

8. Surface stress effects in UV-induced phase-state changes

In the manuscript, we have discussed UV-induced morphological changes of DNA gel condensates (the rounding-up of a Y_{3x5} condensate in Fig. 2a(i) and collapse behavior of a $Y_{1x7}/L_0/Y_0$ cross-linked motif condensate in Fig. 5f) in relation to surface stress. Below is provided Supplementary Fig. 7 for a graphical understanding of the surface stress-related deformations.

Generally, surface stress S is an interfacial force per unit length that acts on the periphery of a finitely deformed surface region (of a length scale L) as a mechanical manifestation of surface energy γ[6,7]. Surface stress S acts to squeeze the deformed surface as surface energy γ favors to minimize the excess surface area, giving rise to a competition with elastic modulus E[8,9]. Thus, the significance of S relative to E dictates the surface stress-related deformation. Its possibility depends on a length scale of interest L in comparison to an elastocapillary length L_C defined as $L_C = S/E$: only when $L \ll L_C$, the interfacial deformation occurs. Surface stress S and surface energy γ are related via Shuttleworth equation[6,7]

$$S = \gamma + \frac{\mathrm{d}\gamma}{\mathrm{d}\varepsilon}, \quad (1)$$

where ε refers to an interfacial strain. Specifically, only in the case of liquids, this equation reduces to $S = \gamma$, as γ is constant throughout the deformation-induced strain changes[7]. This liquid-state property is typically referred to as surface tension or surface energy interchangeably. In the case of solids or gels, however, the strain-induced molecular structure depends on a strain applied. This ε dependency amounts to $S \neq \gamma$. In this study, we prefer the use of surface "stress" to surface "tension" as the irradiated condensates phase-transition between (soft) gel and liquid states.

In a two-phase system comprising a Y_{3x5} gel-state condensate and surrounding water (serving as a buffer) as illustrated in Supplementary Fig. 7a, the condensate (with an modulus E_{3x5}) feels a surface stress $S_{3x5\text{–}W}$ at the interface. In the initial state as a soft solid, large E_{3x5} overwhelms $S_{3x5\text{–}W}$, suppressing the surface stress deformation. With UV irradiation, the constituting motifs build up mobility (as shown in the FRAP experiments, Fig. 2d), giving rise to an increase in the condensate fluidity. This in turn reduces the condensate elasticity E_{3x5} to E'_{3x5}, and accordingly increases the elastocapillary length L_C to L'_C. Consequently, a wider range of the length scales falls within a regime of $L \ll L'_C$. Given no drastic scale change in the condensate size, surface stress-related effects become more prominent across the condensate surface. Surface energy $\gamma_{3x5\text{–}W}$ comes into play to minimize the surface-to-volume ratio as an energy penalty on the surface. This amounts to a large rounding-up of the irregular shape in the gel-to-liquid phase-transitioning Y_{3x5} condensate.

In the molecular viewpoint, the increased fluidity of DNA condensates can be attributed to enhanced reshuffling among the constituting motifs, where their interchangeable dissociation and association lead to an increased macroscopic fluidity[10]. In addition, we believe that the density of the gel-to-liquid phase-transitioned condensates should have remained almost invariant throughout the photoinduced morphological change. The surface energy γ only acted to reduce the surface against the volume occupied by the state-transitioning condensates.

Next, another step-by-step account is given of the UV-induced collapse in the $Y_{1x7}/L_0/Y_0$ cross-linked motif condensate (Supplementary Fig. 7b). In the initial state, the two immiscible phases, Y_{1x7} and Y_0 condensates, are in a gel state, both with elastic moduli E_{1x7} and E_0, respectively. The cross-linker L_0 allows these phases to possess a high binding affinity. At the interfacial boundaries, where the three phases meet, there is a force balance that involves surface stresses $S_{1x7\text{–}W}$, $S_{1x7\text{–}0}$, and $S_{0\text{–}W}$ (here, the triple-phase boundary is abbreviated as '3PB'). The first step is UV-induced fluidity increase in the Y_{1x7} condensate. By the same token as Supplementary Fig. 7a, Y_{1x7} condensate favors to round up from the irregular shape. As the surface stress $S_{1x7\text{–}0}$ becomes more relevant at the Y_{1x7} and Y_0 interfaces, the force balance between $S_{1x7\text{–}W}$, $S_{1x7\text{–}0}$, and $S_{0\text{–}W}$ at the 3PB experiences rebalancing.

Due to the high binding affinity, Y_{1x7} condensate energetically favors increased interfacial areas with Y_0 condensates, a phenomenon known as wetting. Lastly, combined with large deformability of the soft Y_0 condensates, this wetting allows the loosely connected Y_0 condensates to self-fold into a smaller size than the initial size. It should be noted that these processes, described in a stepwise manner, co-occur.

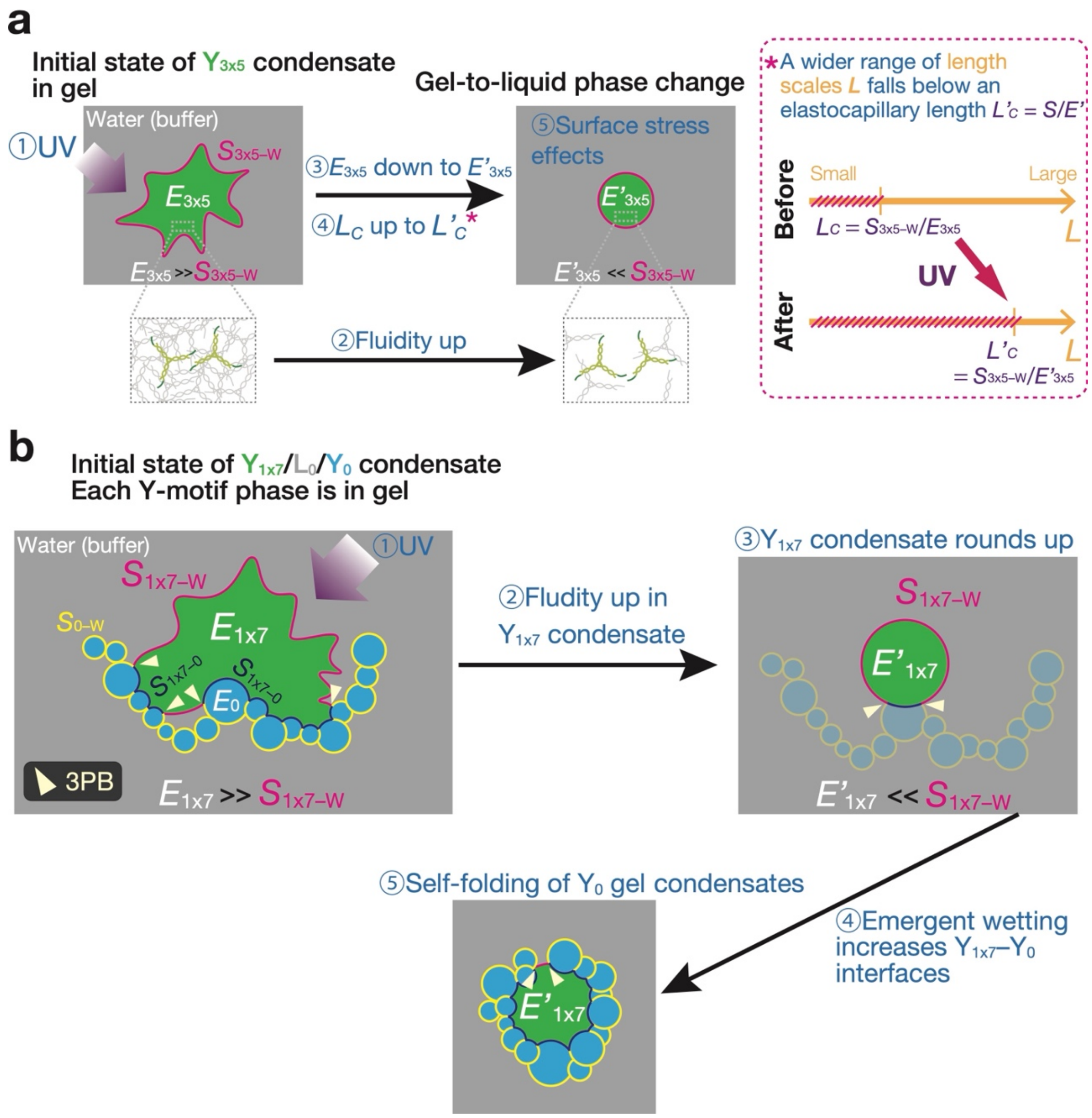

Supplementary Fig. 7 Step-by-step depiction of surface stress effects in UV-induced phase-state changes. a, Schematics of a rounding-up of a UV-irradiated Y_{3x5} gel-state condensate interfaced with surrounding water (as a buffer). At the interface, surface stress $S_{3x5\text{–W}}$ is present in a competition with elastic modulus E_{3x5}. With UV irradiation, E_{3x5} reduces to E'_{3x5} as a result of fluidity increase. L_c refers to an elastocapillary length, defined as S/E, below which surface stress-related condensate

deformation occurs. **b,** Illustration of self-folding in a UV-irradiated $Y_{1x7}/L_0/Y_0$ gel-state condensate. Cross-linker L_0 serves as an adhesive that combines immiscible Y_{1x7}/Y_0 phases, in green and blue, respectively. Surface stresses $S_{1x7\text{-W}}$ (magenta), $S_{1x7\text{-}0}$ (navy), and $S_{0\text{-W}}$ (yellow) act on the interfaces of Y_{1x7}–water, Y_{1x7}–Y_0, Y_0–water, respectively. These surface stresses are balanced at the triple-phase boundaries (labeled as 3PBs).

9. Measurement of MSD

Time-series images of photo-generated migrations of Y_0 particles were analyzed using TrackMate, an open-source plugin in Fiji, to quantify their mobility. The plugin allowed us to select captured trajectories whose duration should span the whole observation time. The recording spanned a time period from turning on the excitation filter to the equilibration of Y0 particles' motion.

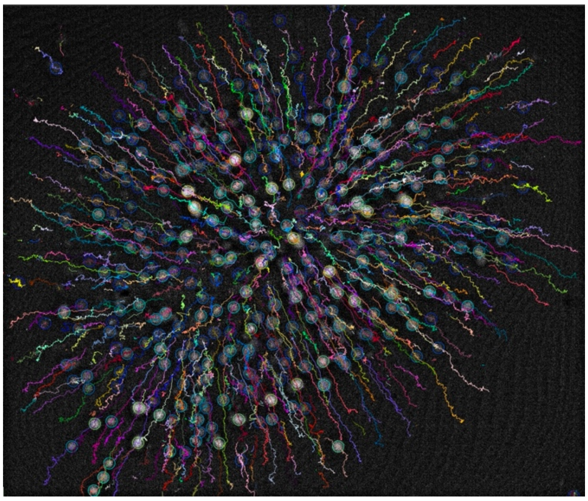

Supplementary Fig. 8 Representative screen capture of the particle tracking using TrackMate. Detected trajectories of the "spread" motion of non-photoresponsive gel particles (outward flow) in $Y_{2x1x5}/L_0/Y_0$ at RT. Paths are colored according to the trajectory IDs. Circles are the LoG detector, a particle tracker in TrackMate.

10. Evaluation of flow mobility from MSD

The means square displacement (MSD) of a Brownian particle motion is related to diffusion coefficient D via $F(\tau) = 4D\tau$ (τ: time interval). In contrast, the ballistic behavior of non-Brownian motion such as self-propulsion and convective transport gives MSD with an upward curve

$$G(\tau) = 4D\tau + \frac{v^2\tau_R^2}{2}\left(\frac{2\tau}{\tau_R} + e^{-\frac{2\tau}{\tau_R}} - 1\right), \qquad (2)$$

where v is the velocity, and τ_R^{-1} is the rotational diffusion coefficient[11,12]. At sufficiently large τ, a linear curve fitting approximates the MSD curve to a linear form of

$$H(\tau) = (4D + v^2\tau_R)\tau - \frac{v^2\tau_R^2}{2}. \tag{3}$$

Herein, we rewrite $D + v^2\tau_R/4$ as an effective diffusion coefficient D' that accounts for enhanced diffusion due to the ballistic behavior of non-Brownian motion. In the current study, D' serves as an indicator of the mobility of photogenerated flow (Fig. 6e).

11. Enthalpy estimation of Azo-modified SEs

In the main manuscript, we have reported the dissolving temperature T_D of photoreactive DNA condensates for evaluating the binding strength of their SEs (Fig. 3). T_D reflects the thermodynamic parameters of the SEs binding strength, as shown in Supplementary Fig. 9. The bar plot presents measured T_D values of various condensates composed of different Azo-free Y-motifs. We adapted the SE of non-Azo Y-motif (Supplementary Table 1) to alter the binding strength, while leaving the stem sequences unchanged (Supplementary Table 11–Supplementary Table 17). We also calculated the thermodynamic parameters of the various non-Azo SEs by using MELTING5[13], a numerical platform for nucleic acids[7], and overlaid them with the bar plot. ΔH, ΔS, and ΔG correspond to the differences of the enthalpy, entropy, and free energy between the bound and unbound states of the paired SEs, respectively. Each of these parameters monotonically relates to T_D.

To describe the underlying mechanism of the multi-mode flow behavior depending on the Azo insertion sites, we have focused our discussion on ΔH for two reasons. First, ΔS is virtually unavailable to obtain for complex network-forming condensates, because ΔS relates to the structural properties (such as freedoms of translation, rotation, and bending). Second, our method of Azo-based photoswitching is limited to direct control of the SEs' binding affinity, *i.e.*, ΔH. Thus, the discussion in the main manuscript highlights ΔH to explain the underlying mechanism behind the multi-mode flow behavior.

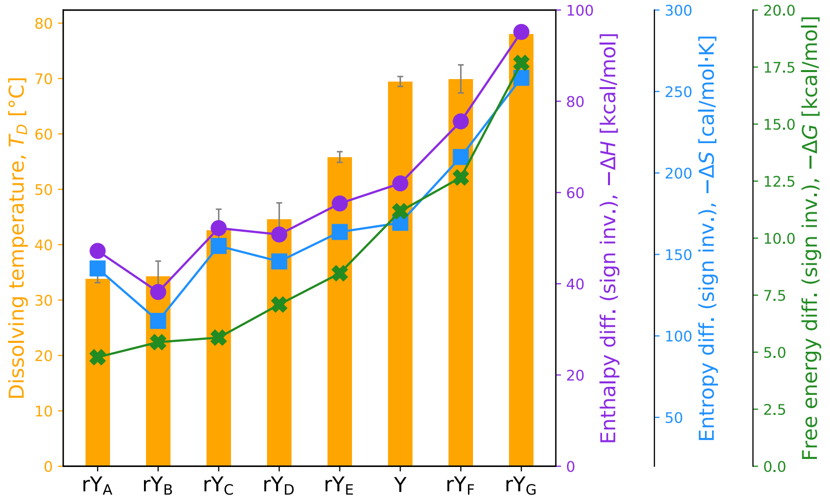

Supplementary Fig. 9 Dissolving temperatures T_D and their relation to thermodynamic parameters for DNA condensates with non-modified SEs. ‘Y’ refers to a Y-motif without Azo insertion (Supplementary Table 1), while others refer to non-modified Y-motifs listed above (Supplementary Table 11–Supplementary Table 17). Error bars: S.D. ($n = 3$). Source data are provided as Source Data files.

Thermodynamically, the observed peak shift (between $Y_{3x5}/L_0/Y_0$ and $Y_{2x1x5}/L_0/Y_0$) and sign inversion (between $Y_{3x5}/L_0/Y_0$ and $Y_{1x7}/L_0/Y_0$) in the temperature dependence of flow mobility D^* (Fig. 6f) can be associated with the enthalpy difference of the SE binding states. This means the difference between the enthalpy of the bound state of SEs and that of the unbound state of the SEs at equilibrium (Supplementary Fig. 10a). Here, we introduce a phase diagram that spans the $|\Delta H|$–T space (Supplementary Fig. 10b), where ΔH indicates the enthalpy difference between the bound and unbound states in the SEs (Supplementary Fig. 10a). For photoresponsive DNA condensates, the bound–unbound enthalpy difference depends significantly on the isomeric state of Azo residues introduced in the SEs: *trans*-Azo and *cis*-Azo enhance the stacking and unstacking of the SEs, respectively, providing a photoswitching mechanism in the bound–unbound enthalpy difference[14,15]. UV irradiation promotes the *trans*-to-*cis* isomerization of Azo and a dynamic increase in ΔH by the amount of $\Delta(\Delta H)_{trans\to cis}$[15,16], whereas Vis causes a *cis*-to-*trans* isomerization accompanied by $-\Delta(\Delta H)_{trans\to cis}$. Thus, $|\Delta H|$ corresponds to the binding strength of the SEs. In the $|\Delta H|$–T phase diagram, the photocontrolled SE switching can be described as an isomerization-guided pathway, which is in the form of a downward and upward vertical shift (Supplementary Fig. 10b). The isomerization pathway causes no change in the phase properties in a very low temperature range (L) since the gel–liquid phase boundary is distant. Similarly, no significant change at very high temperatures (H) is observed in the isomerization since the path is below the liquid–dissociated

boundary. However, the isomerization pathway can cross the boundary only within the intermediate temperature window (M), which leads to a significant fluidity change via the macroscopic phase transition.

Based on the $|\Delta H|-T$ phase map, the observed horizontal shift (between $Y_{3x5}/L_0/Y_0$ and $Y_{2x1x5}/L_0/Y_0$) and drastic sign inversion (between $Y_{3x5}/L_0/Y_0$ and $Y_{1x7}/L_0/Y_0$) of the D^* profiles (Fig. 6f) can be explained using the vertical length of the isomerization path $\Delta(\Delta H)$ within the intermediate temperature window. To evaluate ΔH in SE_{1x7}, SE_{3x5}, and SE_{2x1x5} possessing *trans/cis* Azo, we measured T_D of various DNA condensates with Azo-free SEs (Supplementary Fig. 10c). While some widely used prediction models[17,18] can readily calculate the thermodynamic parameters of unmodified DNA sequences, there is no well-established model that predicts those of Azo-modified DNA sequences[13,15]. However, the thermodynamic parameters of the unmodified SEs were monotonically correlated with the measured T_D of the resulting DNA condensates (Supplementary Fig. 9). This monotonicity between $|\Delta H|$ in the Azo-free SEs and T_D of the corresponding condensates means that the indirect estimation of $|\Delta H|$ for the Azo-modified SEs is possible by measuring T_D of the resulting condensates. We drew a fitting curve on the data points obtained for the measured T_D of Azo-free condensates in a range from RT up to 60°C (Supplementary Fig. 10c). By applying this fitting curve to the measured T_D for Azo-modified SEs, we obtained $|\Delta H|$ estimates for SE_{1x7} (*trans/cis*), SE_{3x5} (*trans*), and SE_{2x1x5} (*cis*), which were well in the fitting range (Supplementary Fig. 10d). Although the $|\Delta H|$ values for *cis* SE_{3x5} and *cis* SE_{2x1x5} fell below the lower limit of the fitting range, the monotonic $T_D-|\Delta H|$ correlation enabled us to estimate these $|\Delta H|$ values. From these $|\Delta H|$ estimations, we deduced an increase of $|\Delta(\Delta H)_{trans\rightarrow cis}|$ in the order of SE_{1x7}, SE_{3x5}, and SE_{2x1x5}.

Now, we reflect the quantified $|\Delta(\Delta H)|$ as a function of the Azo insertion site in the phase map to explain the observed sign inversion and shift in D^* of the resulting microflow. Supplementary Fig. 10e shows that the insertion site in the SE defines a reaction pathway of length $|\Delta(\Delta H)_{trans\rightarrow cis}|$ in the $|\Delta H|-T$ phase diagram. For $Y_{1x7}/L_0/Y_0$ with the lowest $|\Delta(\Delta H)_{trans\rightarrow cis}|$, the *trans*-to-*cis* isomerization path can cross the gel-to-liquid phase boundary but not as far as the liquid-to-dissociated phase boundary. This moderate photogenerated $|\Delta(\Delta H)_{trans\rightarrow cis}|$ produced the pronounced fluid effect, *i.e.,* the inward-directed large collapse of Y_0 gel particles (Fig. 5f). In contrast, for $Y_{3x5}/L_0/Y_0$ and $Y_{2x1x5}/L_0/Y_0$ with a significantly larger $|\Delta(\Delta H)_{trans\rightarrow cis}|$, the isomerization paths were long enough to cross the liquid–dissociated boundary, and favored a gel-to-dissociated state transition, which was a driving force for the outward-directed spread mode (Fig. 5d. The sign inversion in the D^* profiles). Furthermore, $Y_{2x1x5}/L_0/Y_0$, possessing a higher $|\Delta(\Delta H)_{trans\rightarrow cis}|$ than $Y_{3x5}/L_0/Y_0$, can extend its pathway further downward, resulting in a marked downshift of the temperature window for the pronounced gel-to-dissociated phase transition.

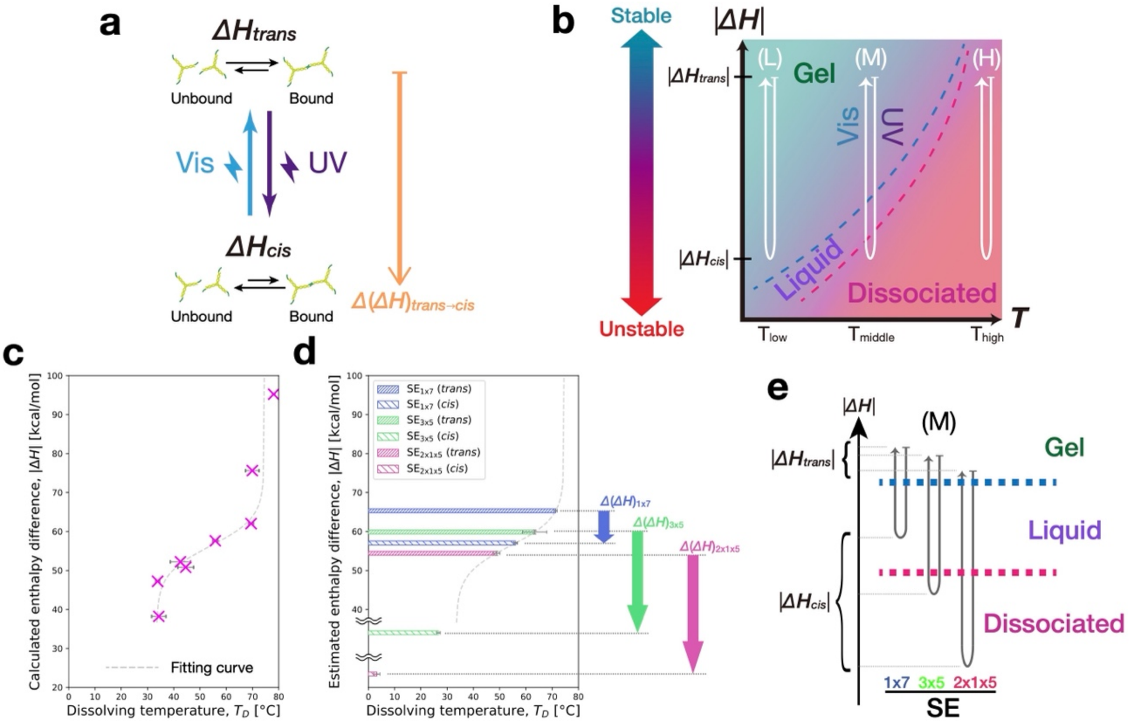

Supplementary Fig. 10 Dynamic enthalpy fine-tuning of SE binding as an underlying mechanism of mode-switchable flow behaviors. **a**, Schematic of the enthalpy difference between the bound and unbound states of two motifs ΔH, which varies according to the isomer of the inserted Azo residues. UV irradiation generates a shift in ΔH by an amount of $\Delta(\Delta H)_{trans \to cis} = \Delta H_{cis} - \Delta H_{trans}$. **b**, Phase diagram that spans the $|\Delta H|$–T space. $|\Delta H|$ corresponds to the binding strength of SEs. Isomerization pathways of SEs for UV irradiation are added in (L) low, (M) middle, and (H) high temperature ranges. **c**, Measured T_D values of DNA condensates with various non-Azo SEs are related to $|\Delta H|$ of the corresponding SEs calculated using MELTING5[13]. Curve fitting was applied to the obtained data points using the form of logit function $f(x) = \ln\{(x-b)/[k-(x-b)]\}^{1/a} + c$. Error bars: S.D. ($n = 3$). **d**, Estimation of $|\Delta H|$ for Azo-modified SE_{1x7} (*trans*, *cis*), SE_{3x5} (*trans*), and SE_{2x1x5} (*cis*). Measured T_D of the condensates (Fig. 3) gives an estimate of their corresponding $|\Delta H|$ in the SE binding using the fitting function in **c**. Vertical error bars denote propagated errors $|f'(x)\sigma|$ (σ: S.D. of T_D in Fig. 3). **e**, Azo insertion site in the SE determines the magnitude of a dynamic enthalpy shift $|\Delta(\Delta H)_{trans \to cis}|$ and a consequent specific flow mode. Source data are provided as Source Data files.

12. Directional motion of DNA liquid-state condensates subjected to localized photoswitching

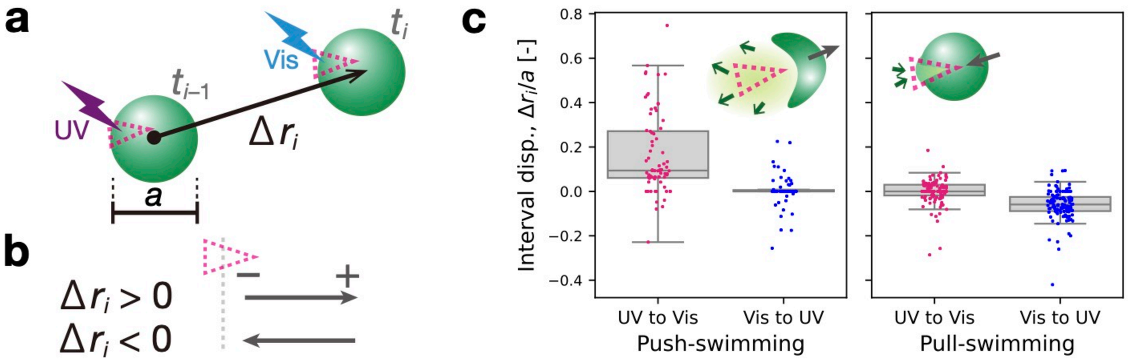

Supplementary Fig. 11 Interval displacement of DNA liquid condensates between photoswitching moments. a, Representative illustration of an interval displacement Δr_i between a UV irradiation at t_{i-1} and the UV-to-Vis switching at t_i, referred to as 'UV to Vis', for a liquid condensate with an initial diameter a. **b,** Sign of Δr_i, defined as positive if the stepwise motion is distanced away from the ROI. **c,** Box plots of interval displacements Δr_i normalized by liquid condensate sizes a for the two types of intervals in (left) push- and (right) pull-swimming, overlaid with the individual data points from the observed directional motion in Fig. 7h. Central lines mark the medians; boxes represent the Q1 (first quartile)–Q3 (third quartile) interquartile range (IQR); whiskers encompass the data points within Q1 – 1.5 × IQR and Q3 + 1.5 × IQR.

13. Numerical experiments of the directional motion in localized photoswitching

In the main manuscript, we have shown the numerical experiments to roughly estimate the one-directional (1D) motions of a photoreactive DNA liquid condensate (Fig. 8). Here, we explain the numerical model and its governing equations (Supplementary Fig. 12).

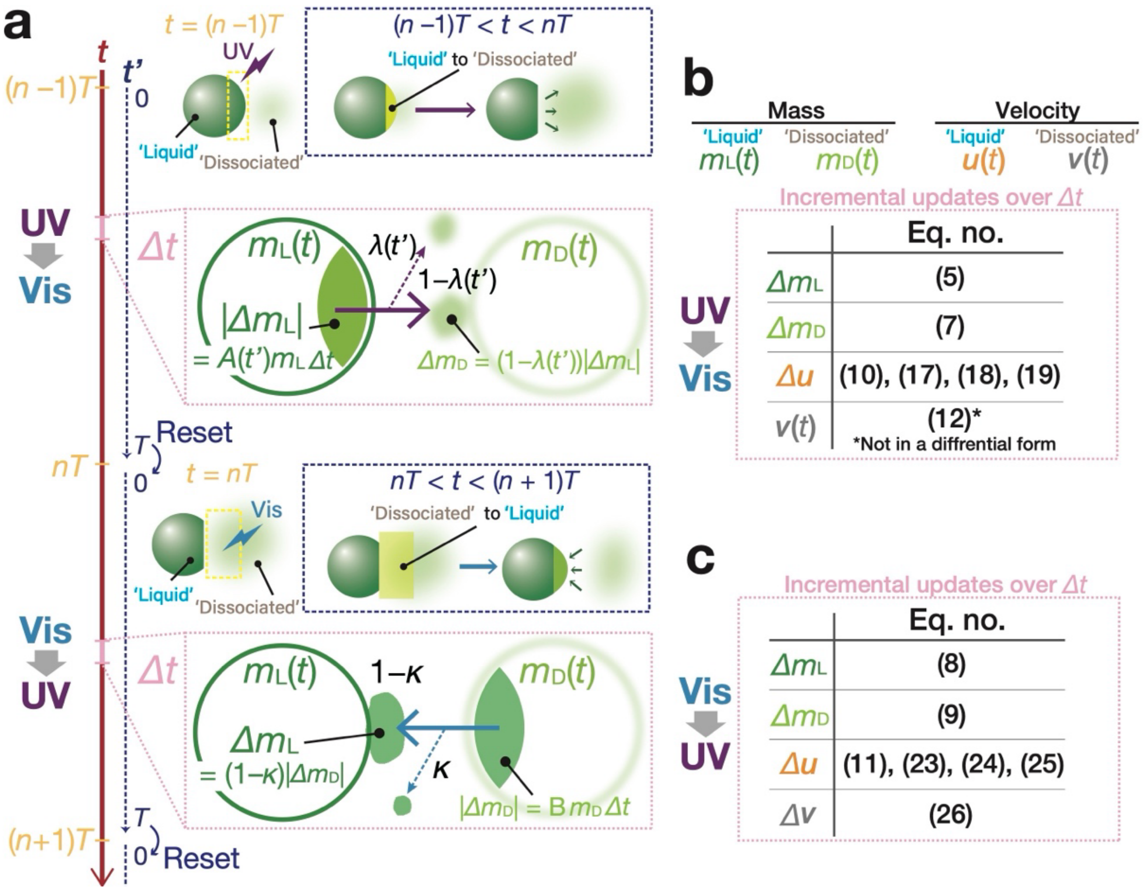

Supplementary Fig. 12 Numerical model simulating the 1D motion of a liquid-state DNA condensate locally photoswitched. The model treats mass and energy transport between 'Liquid'- and 'Dissociated'-state domains in the condensate. **a,** Representative sketch of the mass transport between the binary states within n^{th} interval (UV-to-Vis) and the following Vis-to-UV interval, each with a time duration of T. Within each interval, a lap time t' ticks through from zero to T, and is reset to zero upon entering the next interval. Localized irradiation is modeled as a pulse-like shot with a negligibly short duration. Dashed-line boxes (in navy) highlight a photoaffected fraction in the condensate that generates UV-discharged microflow and Vis-reversed flow over an interval of T, respectively. Dotted-line boxes (in pink) depict the corresponding mass transport over a time step Δt. The model performs incremental updating of time-dependent variables for **b** UV-to-Vis intervals and **c** Vis-to-UV intervals. Their incremental alterations over Δt are obtained using the corresponding differential equations, with the reference numbers listed in the tables.

Consider a DNA liquid condensate subjected to photoswitching localized within a region-of-interest (ROI), as depicted in Supplementary Fig. 12a. The constituting motifs undergo transport between 'Liquid'- and 'Dissociated'-state domains with the time-dependent masses, $m_{\mathrm{L}}(t)$ and $m_{\mathrm{D}}(t)$, respectively. In the model, UV and Vis irradiations were idealized to be of a pulse-like profile with an infinitesimally short duration for simplicity. Upon UV irradiation, 'Liquid'-state domain specified within an ROI yields to a spreading flow during a UV-to-Vis interval with a time length T. In this interval, a fraction $A(t')$ of 'Liquid'-state domain exits in the form of microflow per unit time, where t' is a lap time elapsed in an interval (UV-to-Vis or Vis-to-UV). Note that t' is reset to zero upon a photoswitching event. Considering intense diffusion effects during UV-to-Vis intervals, the disassembly ratio $A(t')$ per unit time was supposed to grow non-linearly with time relative to a characteristic diffusion time τ^* as

$$A(t') = A_0(t'/\tau^*)^{3/2}, \tag{4}$$

where A_0 is constant. The exponent 3/2 reflects the well-known relationship $\langle r^2(\tau)\rangle \sim 2dD\tau$ (d: dimension) between mean square displacement $\langle r^2(\tau)\rangle$ and diffusion coefficient D. The UV-generated microflow exit results in an incremental change of 'Liquid'-state mass $m_{\mathrm{L}}(t)$, given by $\Delta m_{\mathrm{L}} = (\mathrm{d}m_{\mathrm{L}}(t)/\mathrm{d}t)\Delta t$ (Δt: time step), where

$$\frac{\mathrm{d}m_{\mathrm{L,\,UV\to Vis}}(t)}{\mathrm{d}t} = -A(t')m_{\mathrm{L,\,UV\to Vis}}(t). \tag{5}$$

Note that $m_{\mathrm{L,\,UV\to Vis}}(t)$ and $m_{\mathrm{L,\,Vis\to UV}}(t)$ accompanied by the subscripts point to the same parameter $m_{\mathrm{L}}(t)$ during UV-to-Vis and Vis-to-UV intervals, respectively. This subscript rule applies to the other parameters. We also reasoned that a fraction of the discharged motifs being transferred to 'Dissociated'-state would be diffused away from the UV–Vis cyclic reactions. This diffusion-associated disengagement ratio $\lambda(t')$ is given by the similar form to Eq.(4):

$$\lambda(t') = \lambda_0(t'/\tau^*)^{3/2}, \tag{6}$$

where λ_0 is constant. Over a time step Δt during UV-to-Vis intervals, 'Dissociated'-state domain with $m_{\mathrm{D}}(t)$ gains an amount of $\Delta m_{\mathrm{D}} = (\mathrm{d}m_{\mathrm{D}}(t)/\mathrm{d}t)\Delta t$, where

$$\frac{\mathrm{d}m_{\mathrm{D,\,UV\to Vis}}(t)}{\mathrm{d}t} = (1-\lambda(t'))A(t')m_{\mathrm{L,\,UV\to Vis}}(t). \tag{7}$$

In contrast, the opposite processes from 'Dissociated'- to 'Liquid'-state domains during Vis-to-UV intervals were presumed to be less susceptible to diffusion effects due to the recondensation. Thus, the Vis-to-UV counterparts, B (reassembly ratio) and κ (disengagement ratio), were regarded as constant. The incremental changes per unit time of 'Liquid' and 'Dissociated' masses in Vis-to-UV intervals are provided by differential equations

$$\frac{\mathrm{d}m_{\mathrm{L,\,Vis\to UV}}(t)}{\mathrm{d}t} = (1-\kappa)Bm_{\mathrm{D,\,Vis\to UV}}(t), \tag{8}$$

$$\frac{\mathrm{d}m_{\mathrm{D,\,Vis\to UV}}(t)}{\mathrm{d}t} = -Bm_{\mathrm{D,\,Vis\to UV}}(t). \tag{9}$$

Next, 1D velocities for 'Liquid'-state domain $u(t)$ are subjected to incremental changes during UV-to-Vis and Vis-to-UV intervals, $\Delta u_{\mathrm{UV\to Vis}}$ and $\Delta u_{\mathrm{Vis\to UV}}$, over a time step Δt. The sign of the 1D velocity is defined as positive when directed away from the ROI (Supplementary Fig. 11a, b). The incremental changes of the velocities are divided into two contributions, one from momentum exchange between the binary states and the other from thermal dissipation:

$$\Delta u_{\mathrm{UV\to Vis}} = \Delta u^{M}_{\mathrm{UV\to Vis}} + \Delta u^{T}_{\mathrm{UV\to Vis}}, \tag{10}$$

$$\Delta u_{\mathrm{Vis\to UV}} = \Delta u^{M}_{\mathrm{Vis\to UV}} + \Delta u^{T}_{\mathrm{Vis\to UV}}, \tag{11}$$

where the superscripts denote the momentum exchange (*M*) and thermal dissipation (*T*). For incremental evaluation of the velocities of 'Liquid'-state domain, those of 'Dissociated'-state domain $v(t)$ are given by the following:

$$v_{\mathrm{UV\to Vis}}(t) = u_{\mathrm{UV\to Vis}}(t) + v_{\mathrm{out}}, \tag{12}$$

$$\Delta v_{\mathrm{Vis\to UV}} = \Delta v^{T}_{\mathrm{Vis\to UV}}. \tag{13}$$

Note that Eq. (12) does not take a differential form. In UV-to-Vis intervals, the model presumes that UV-affected motifs are discharged from migrating 'Liquid'-state domain with a constant velocity of v_{out} defined as $v(t)_{\mathrm{UV\to Vis}} - u(t)_{\mathrm{UV\to Vis}} = v_{\mathrm{out}}$ (a negatively signed constant). Since the UV-discharged microflow is distanced away from the 'Liquid'-state domain, v_{out} is a negatively signed constant. This velocity corresponds to that of the outward-directed spreading microflow under isotropic UV irradiation as seen in Fig. 5g. We reasonably neglected the thermal dissipation effect in the previous time steps. This simplification can be justified by the constant supply of UV-discharged fractions from 'Liquid' to 'Dissociated' domains, the latest segment of which joins the reverse transfer in the upcoming Vis-to-UV interval. Meanwhile, the incremental velocity updating during Vis-to-UV intervals in Eq. (13) features the unaffected segment of 'Dissociated'-state domain that remains subjected to thermal dissipation.

For the velocities of 'Liquid' domain in UV-to-Vis intervals (Eq. (10)), while setting aside the dissipation term $\Delta u^{T}_{\mathrm{UV\to Vis}}$, the momentum exchange-associated $\Delta u^{M}_{\mathrm{UV\to Vis}}$ over a time step Δt was derived from the following conservation equation:

$$u^{M}_{\mathrm{UV\to Vis}} m_{\mathrm{L,\,UV\to Vis}} = (u^{M}_{\mathrm{UV\to Vis}} + \Delta u^{M}_{\mathrm{UV\to Vis}})\big(m_{\mathrm{L,\,UV\to Vis}} + \Delta m_{\mathrm{L}}\big) + (u^{M}_{\mathrm{UV\to Vis}} + v_{\mathrm{out}})(-\Delta m_{\mathrm{L}}). \tag{14}$$

Note that this conservation equation concerns an idealized non-dissipative velocity $u^{M}_{\mathrm{UV\to Vis}}$ for simplicity. With $\Delta u^{M}_{\mathrm{UV\to Vis}} = (\mathrm{d}u^{M}_{\mathrm{UV\to Vis}}(t)/\mathrm{d}t)\Delta t$ and $\Delta m_{\mathrm{L,\,UV\to Vis}} = (\mathrm{d}m_{\mathrm{L,\,UV\to Vis}}(t)/\mathrm{d}t)\Delta t$, one may neglect second-order $\Delta u^{M}_{\mathrm{UV\to Vis}}\,\Delta m_{\mathrm{L}}$, reducing Eq. (14) to

$$\Delta u^{M}_{\mathrm{UV\to Vis}}\, m_{\mathrm{L,\,UV\to Vis}} = \Delta m_{\mathrm{L}} v_{\mathrm{out}}. \tag{15}$$

In the differential form,

$$\frac{\mathrm{d}u^{M}_{\mathrm{UV\to Vis}}(t)}{\mathrm{d}t}=\frac{1}{m_{\mathrm{L,\,UV\to Vis}}}\frac{\mathrm{d}m_{\mathrm{L,\,UV\to Vis}}(t)}{\mathrm{d}t}v_{\mathrm{out}}. \tag{16}$$

From Eq. (5), we find

$$\frac{\mathrm{d}u^{M}_{\mathrm{UV\to Vis}}(t)}{\mathrm{d}t}=-A(t')v_{\mathrm{out}}, \tag{17}$$

from which $\Delta u^{M}_{\mathrm{UV\to Vis}}=-A(t')v_{\mathrm{out}}\Delta t$ in Eq. (10). Further, in the presence of thermal dissipation, an overdamped system is relevant, where viscous forces dominate. They oppose the migration with a velocity $u_{\mathrm{UV\to Vis}}(t)$. Thus, we obtain $\Delta u^{T}_{\mathrm{UV\to Vis}}=(\mathrm{d}u^{T}_{\mathrm{UV\to Vis}}(t)/\mathrm{d}t)\Delta t$, where

$$\frac{\mathrm{d}u^{T}_{\mathrm{UV\to Vis}}(t)}{\mathrm{d}t}=-\frac{C_T}{m_{\mathrm{L,\,UV\to Vis}}(t)}u_{\mathrm{UV\to Vis}}(t), \tag{18}$$

(C_T: a thermal dissipation coefficient). Substituting Eqs. (17) and (18) into Eq. (10), we find incremental velocity changes during UV-to-Vis intervals $\Delta u_{\mathrm{UV\to Vis}}(t)=(\mathrm{d}u_{\mathrm{UV\to Vis}}(t)/\mathrm{d}t)\Delta t$, where

$$\frac{\mathrm{d}u_{\mathrm{UV\to Vis}}(t)}{\mathrm{d}t}=\frac{\mathrm{d}u^{M}_{\mathrm{UV\to Vis}}(t)}{\mathrm{d}t}+\frac{\mathrm{d}u^{T}_{\mathrm{UV\to Vis}}(t)}{\mathrm{d}t}=-\left(A(t')v_{\mathrm{out}}+\frac{C_T}{m_{\mathrm{L,\,UV\to Vis}}(t)}u_{\mathrm{UV\to Vis}}(t)\right). \tag{19}$$

Similarly, in Vis-to-UV intervals, the momentum exchange-associated $\Delta u^{M}_{\mathrm{Vis\to UV}}$ over a time step Δt was derived from the following, with $\Delta u^{T}_{\mathrm{UV\to Vis}}$ set aside:

$$u^{M}_{\mathrm{Vis\to UV}}\,m_{\mathrm{L,\,Vis\to UV}}+(v_{\mathrm{Vis\to UV}}+v_{\mathrm{in}})\Delta m_{\mathrm{L}}=(u^{M}_{\mathrm{Vis\to UV}}+\Delta u^{M}_{\mathrm{Vis\to UV}})\big(m_{\mathrm{L,\,Vis\to UV}}+\Delta m_{\mathrm{L}}\big). \tag{20}$$

Here, v_{in} refers to an additional velocity of the Vis-reversed fraction being transferred from 'Dissociated'-state domain migrating with a velocity $v_{\mathrm{Vis\to UV}}$. This v_{in}, regarded as constant in this model, corresponds to the velocity of the inward-directed Vis-reversed microflow under isotropic irradiation as seen in Fig. 5h(ii). In the localized irradiation, the Vis-reversed flow is attracted to the ROI. Hence, v_{in} is negatively signed. While neglecting second-order $\Delta u^{M}_{\mathrm{UV\to Vis}}\,\Delta m_{\mathrm{L}}$, this conservation equation reduces to

$$\Delta u^{M}_{\mathrm{Vis\to UV}}\;m_{\mathrm{L,\,Vis\to UV}}=(v_{\mathrm{Vis\to UV}}+v_{\mathrm{in}}-u^{M}_{\mathrm{Vis\to UV}})\Delta m_{\mathrm{L}}, \tag{21}$$

where $\Delta u^{M}_{\mathrm{Vis\to UV}}=(\mathrm{d}u^{M}_{\mathrm{Vis\to UV}}(t)/\mathrm{d}t)\Delta t$ and $\Delta m_{\mathrm{L}}=(\mathrm{d}m_{\mathrm{L,\,Vis\to UV}}(t)/\mathrm{d}t)\Delta t$. By cancelling out Δt, Eq. (21) transforms into the following differential form

$$\frac{\mathrm{d}u^{M}_{\mathrm{Vis\to UV}}(t)}{\mathrm{d}t}=\frac{1}{m_{\mathrm{L,\,Vis\to UV}}(t)}\frac{\mathrm{d}m_{\mathrm{L,\,Vis\to UV}}(t)}{\mathrm{d}t}(v_{\mathrm{Vis\to UV}}(t)+v_{\mathrm{in}}-u^{M}_{\mathrm{Vis\to UV}}(t)). \tag{22}$$

From Eq. (8), this equation is rewritten to

$$\frac{\mathrm{d}u^{M}_{\mathrm{Vis\to UV}}(t)}{\mathrm{d}t}=\frac{(1-\kappa)\,B\,m_{\mathrm{D,\,Vis\to UV}}(t)}{m_{\mathrm{L,\,Vis\to UV}}(t)}(v_{\mathrm{Vis\to UV}}(t)+v_{\mathrm{in}}-u^{M}_{\mathrm{Vis\to UV}}(t)). \tag{23}$$

Meanwhile, the dissipation term $\Delta u^{T}_{\mathrm{Vis\to UV}}$ takes the same form as the UV-to-Vis counterpart $\Delta u^{T}_{\mathrm{UV\to Vis}}$:

$$\frac{\mathrm{d}u^{T}_{\mathrm{Vis}\to\mathrm{UV}}(t)}{\mathrm{d}t} = -\frac{C_T}{m_{\mathrm{L,\,Vis}\to\mathrm{UV}}(t)} u_{\mathrm{Vis}\to\mathrm{UV}}(t). \tag{24}$$

Taking together Eqs. (23) and (24), the velocity increments during Vis-to-UV intervals in Eq. (11) are obtained by $\Delta u_{\mathrm{Vis}\to\mathrm{UV}}(t) = (\mathrm{d}u_{\mathrm{Vis}\to\mathrm{UV}}(t)/\mathrm{d}t)\Delta t$, where

$$\begin{aligned}\frac{\mathrm{d}u_{\mathrm{Vis}\to\mathrm{UV}}(t)}{\mathrm{d}t} &= \frac{\mathrm{d}u^{M}_{\mathrm{Vis}\to\mathrm{UV}}(t)}{\mathrm{d}t} + \frac{\mathrm{d}u^{T}_{\mathrm{Vis}\to\mathrm{UV}}(t)}{\mathrm{d}t} \\ &= \frac{(1-\kappa)\,B\,m_{\mathrm{D,\,Vis}\to\mathrm{UV}}(t)}{m_{\mathrm{L,\,Vis}\to\mathrm{UV}}(t)}\left(v_{\mathrm{Vis}\to\mathrm{UV}}(t) + v_{\mathrm{in}} - u_{\mathrm{Vis}\to\mathrm{UV}}(t)\right) - \frac{C_T}{m_{\mathrm{L,\,Vis}\to\mathrm{UV}}(t)} u_{\mathrm{Vis}\to\mathrm{UV}}(t).\end{aligned} \tag{25}$$

Importantly, it should be reminded that $v_{\mathrm{Vis}\to\mathrm{UV}}(t)$, the current velocity of 'Dissociated' domain, originates from the UV-discharged microflow directed away from 'Liquid' domain in the previous UV-to-Vis interval. This means that with decreasing a Vis-to-UV interval, 'Liquid' domain can gain a more conserved amount of the oppositely directed momentum which 'Liquid' domain has produced in the previous UV-to-Vis interval, in addition to $m_{\mathrm{D,\,Vis}\to\mathrm{UV}}(t)\ v_{\mathrm{in}}$ directed toward the Vis-irradiated ROI.

On the other hand, the dissipation term for the velocity increments of 'Dissociated' domain $\Delta v^{T}_{\mathrm{Vis}\to\mathrm{UV}}$ in Eq. (13) assumes a viscous force that opposes the migration with a velocity $v_{\mathrm{Vis}\to\mathrm{UV}}(t)$, as introduced for the counterpart of 'Liquid' domain in Eq. (24). Hence, $\Delta v_{\mathrm{Vis}\to\mathrm{UV}}(t) = \Delta v^{T}_{\mathrm{Vis}\to\mathrm{UV}} = (\mathrm{d}v^{T}_{\mathrm{Vis}\to\mathrm{UV}}(t)/\mathrm{d}t)\Delta t$, where

$$\frac{\mathrm{d}v_{\mathrm{Vis}\to\mathrm{UV}}(t)}{\mathrm{d}t} = \frac{\mathrm{d}v^{T}_{\mathrm{Vis}\to\mathrm{UV}}(t)}{\mathrm{d}t} = -C_{MT} v_{\mathrm{Vis}\to\mathrm{UV}}(t). \tag{26}$$

Here, C_{MT} refers to a thermal dissipation coefficient for 'Dissociated' domain, which remains unaffected during Vis-to-UV intervals. Note that the size effect of the domain is neglected as opposed to the counterpart of 'Liquid' domain in Eq. (24), because of no distinct boundary for 'Dissociated'-state domain. Thus, the coefficient C_{MT} relates to molecular-level thermal dissipation effects.

The simulations were initiated with the following input values: $A_0 = 0.2$, $B = 0.8$; $\lambda_0 = 0.2$, $\kappa = 0.05$; $v_{\mathrm{out}} = -0.4$, $v_{\mathrm{in}} = -0.2$; $C_T = 0.4$, $C_{MT} = 0.1$; $\Delta t = 0.01$; $\tau^* = 800\Delta t$. The initial values of the masses, velocities and positions for 'Liquid' and 'Dissociated' domains were: $m_{\mathrm{L}}(0) = 100$, $m_{\mathrm{D}}(0) = 0$; $u(0) = 0$, $v(0) = 0$; $x(0) = 0$. Interval lengths T and corresponding switching frequencies $f = 1/(2T)$ were: $T = 800\Delta t$ ($f = 0.0625$), $T = 400\Delta t$ ($f = 0.125$), and $T = 40\Delta t$ ($f = 1.25$) at low, intermediate, and high switching frequencies, respectively. All these variables were treated as dimensionless in the model.

Below, the effect of the momentum exchange in Vis-to-UV intervals was evaluated (Supplementary Fig. 13). With the same values given above, a comparison was made between the cases with and without the momentum exchange terms at low and higher switching frequencies f, the same as used for the low and intermediate cases in Fig. 8c. The simulated results show that positively signed push-swimming was dominant at the lower and higher f, a categorical elimination of f

dependency in the migration direction. Pull-swimming behavior was not available in the absence of the momentum exchange. This suggests that the energy transfer from ‘Dissociated’ to ‘Liquid’ states was crucial to the pull-swimming. During UV-to-Vis intervals, the ‘Liquid’ domain is propelled by UV-discharged microflow, while consuming its mass m_{L}. Within an elongated UV-to-Vis interval (lower f), ‘Liquid’ domain spends more of the “fuel” on enhancing positively directed swimming, which grows non-linearly with time. A tighter interval (higher f), in contrast, discourages such non-linear growth of propelled swimming. Further, the momentum being transferred from ‘Dissociated’ to ‘Liquid’ states has a more conserved amount of the momentum that has originated in the previous UV-to-Vis interval as negatively signed UV-discharged microflow. The net effect is the appearance of the microflow pulling ‘Liquid’ domain toward the ROI.

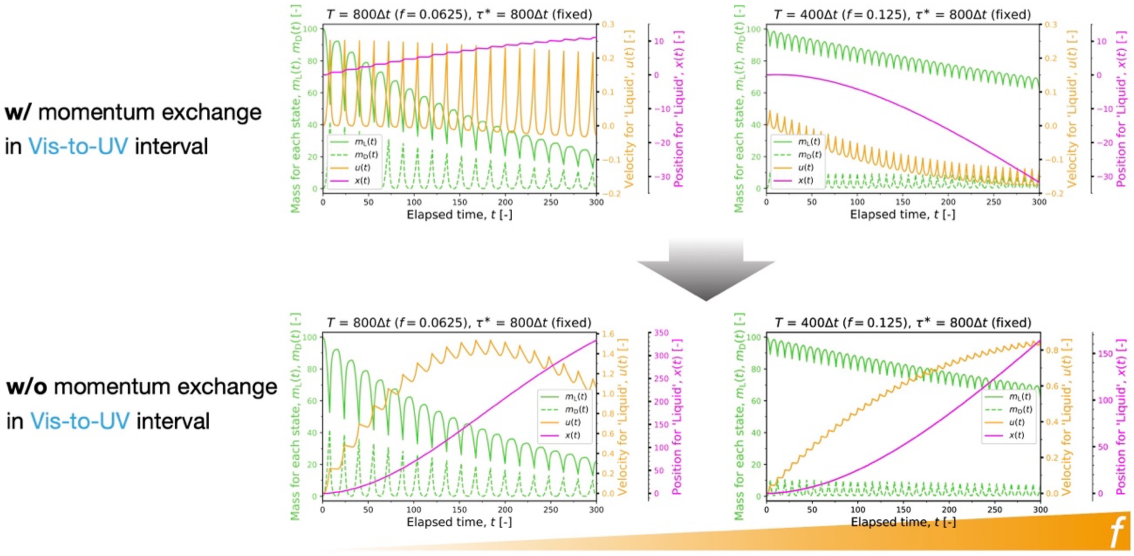

Supplementary Fig. 13 Effect of momentum exchange in Vis-to-UV intervals. Simulated time-evolution of ‘Liquid’/‘Dissociated’ states (top) in the presence of and (bottom) in the absence of momentum exchange between the binary states. The f dependency of the migration direction is eliminated.

Supplementary References

1. Gao, D. *et al.* Controlling the size and adhesion of DNA droplets using surface- enriched DNA molecules. *Soft Matter* (2024) doi:10.1039/d3sm01264f.
2. Griffiths, J. II. Photochemistry of azobenzene and its derivatives. *Chem. Soc. Rev.* **1**, 481 (1972).
3. Fischer, E., Frankel, M. & Wolovsky, R. Wavelength Dependence of Photoisomerization Equilibria in Azocompounds. *J. Chem. Phys.* **23**, 1367–1367 (1955).
4. Beharry, A. A. & Woolley, G. A. Azobenzene photoswitches for biomolecules. *Chem. Soc. Rev.* **40**, 4422–4437 (2011).
5. Nishimura, N. *et al.* Thermal Cis -to- Trans Isomerization of Substituted Azobenzenes II. Substituent and Solvent Effects. *Bulletin of the Chemical Society of Japan* **49**, 1381–1387 (1976).
6. Shuttleworth, R. The surface tension of solids. *Proc. Phys. Soc.* **63**, 444–457 (1950).
7. Andreotti, B. & Snoeijer, J. H. Soft wetting and the Shuttleworth effect, at the crossroads between thermodynamics and mechanics. *EPL* **113**, 66001 (2016).
8. Style, R. W., Jagota, A., Hui, C.-Y. & Dufresne, E. R. Elastocapillarity: Surface tension and the mechanics of soft solids. *Annu. Rev. Condens. Matter Phys.* **8**, 99–118 (2017).
9. Bico, J., Reyssat, É. & Roman, B. Elastocapillarity: When Surface Tension Deforms Elastic Solids. *Annu. Rev. Fluid Mech.* **50**, 629–659 (2018).
10. Cardellini, A. *et al.* Unsupervised data-driven reconstruction of molecular motifs in simple to complex dynamic micelles. *J. Phys. Chem. B* **127**, 2595–2608 (2023).
11. Dunderdale, G., Ebbens, S., Fairclough, P. & Howse, J. Importance of particle tracking and calculating the mean-squared displacement in distinguishing nanopropulsion from other processes. *Langmuir* **28**, 10997–11006 (2012).
12. Howse, J. R. *et al.* Self-motile colloidal particles: from directed propulsion to random walk. *Phys. Rev. Lett.* **99**, 048102 (2007).
13. Dumousseau, M., Rodriguez, N., Juty, N. & Le Novère, N. MELTING, a flexible platform to predict the melting temperatures of nucleic acids. *BMC Bioinformatics* **13**, 101 (2012).
14. Kool, E. T. Preorganization of DNA: Design Principles for Improving Nucleic Acid Recognition by Synthetic Oligonucleotides. *Chem. Rev.* **97**, 1473–1488 (1997).
15. Asanuma, H., Matsunaga, D. & Komiyama, M. Clear-cut photo-regulation of the formation and dissociation of the DNA duplex by modified oligonucleotide involving multiple azobenzenes. *Nucleic Acids Symp. Ser.* 35–36 (2005) doi:10.1093/nass/49.1.35.
16. Dias, A. R. *et al.* Enthalpies of formation of cis-azobenzene and trans-azobenzene. *J. Chem. Thermodyn.* **24**, 439–447 (1992).
17. Borer, P. N., Dengler, B., Tinoco, I., Jr & Uhlenbeck, O. C. Stability of ribonucleic acid double-stranded helices. *J. Mol. Biol.* **86**, 843–853 (1974).

18. SantaLucia, J., Jr, Allawi, H. T. & Seneviratne, P. A. Improved nearest-neighbor parameters for predicting DNA duplex stability. *Biochemistry* **35**, 3555–3562 (1996).
