# Supplementary material for: Remote-controlled mechanical and directional motions of photoswitchable DNA condensates
Source: Nat Commun. 2025 May 14;16:4479. doi: 10.1038/s41467-025-59100-x (PMC12078559; doi:10.1038/s41467-025-59100-x)
Supplement: Supplementary file 3 — Description of Additional Supplementary Files [file 41467_2025_59100_MOESM3_ESM.pdf]

**Description of Additional Supplementary Files**

File Name: Supplementary Movie 1
Description: UV-induced gel-to-liquid state transition of DNA condensates

File Name: Supplementary Movie 2
Description: Vis-induced dissociated-to-liquid state transition of DNA condensates

File Name: Supplementary Movie 3
Description: UV-induced “spread” mode in adhered DNA condensates

File Name: Supplementary Movie 4
Description: UV-induced “collapse” mode in adhered DNA condensates

File Name: Supplementary Movie 5
Description: “Spread and collect” mode under alternating UV/Vis irradiation

File Name: Supplementary Movie 6
Description: Decreasing reversibility of the “spread and collect” cycles

File Name: Supplementary Movie 7
Description: Push-swimming of a DNA liquid condensate in localized UV–Vis photoswitching

File Name: Supplementary Movie 8
Description: Pull-swimming of a DNA liquid condensate in localized UV–Vis photoswitching

File Name: Supplementary Movie 9
Description: Cargo transport using the push-swimming

File Name: Supplementary Movie 10
Description: Cargo transport using the pull-swimming
